# Complete nicotine biosynthesis reshapes the understanding of a classic alkaloid metabolic pathway

**DOI:** 10.3389/fpls.2026.1860687

**Published:** 2026-05-19

**Authors:** Jieling Hong, Xiaoxu Li, Zhen Ma, Zhe Zhao, Zhe Zhang, Daozhu Dong, Kejun Zhong, Wei Luo, Bo Kong, Zhiyuan Li

**Affiliations:** 1 Technology Center, China Tobacco Hunan Industrial Co., Ltd., Changsha, China; 2 Tobacco Research Institute, Chinese Academy of Agricultural Sciences, Qingdao, China; 3 Key Laboratory of Biosynthesis and Biomanufacturing in Model Plants (Beijing Life Science Academy), Ministry of Industry and Information Technology, Beijing, China; 4 Technology Center, China Tobacco Shandong Industrial Co., Ltd., Jinan, China; 5 China Tobacco Hebei Industrial Co., Ltd., Shijiazhuang, China

**Keywords:** BBL proteins, ERF, metabolic engineering, MYC2, nicotine biosynthesis, tobacco alkaloids, transport and compartmentation

## 1 Introduction

Nicotine, a well-known plant alkaloid, has a biosynthesis process which was not fully understood for decades. Both empirical and theoretical studies in plant physiology demonstrate that nicotine is synthesized through two distinct metabolic branches, including the pyridine branch and the pyrrolidine branch. The pyridine branch is derived from the nicotinamide adenine dinucleotide (NAD+) metabolic pathway, and the pyrrolidine branch is derived from the polyamine metabolic pathway (Dewey and Xie, 2013; Kajikawa et al., 2017). Over recent decades, the biochemical pathways of nicotine biosynthesis have been significantly extended, revealing insights into the primary and secondary structures, along with the intricate positive and negative feedback loops governing key modular control elements such as putrescine N-methyltransferase (PMT), reductase A622, berberine bridge enzyme-like enzymes (BBL), the NIC2-locus ethylene responsive factor (ERF) cluster, myelocytomatosis 2 (MYC2), and their associated transporters (Shoji et al., 2010; Shoji and Hashimoto, 2011; Morita et al., 2009; Kato et al., 2014). However, the nicotine biosynthetic network has been lacking adequate descriptions of the molecules that provides structures to the network complexity, rendering the understanding of the biosynthetic process practically incomplete (Kajikawa et al., 2009, Kajikawa et al., 2011).

## 2 From precursor branches to pathway closure

**Figure 1:**
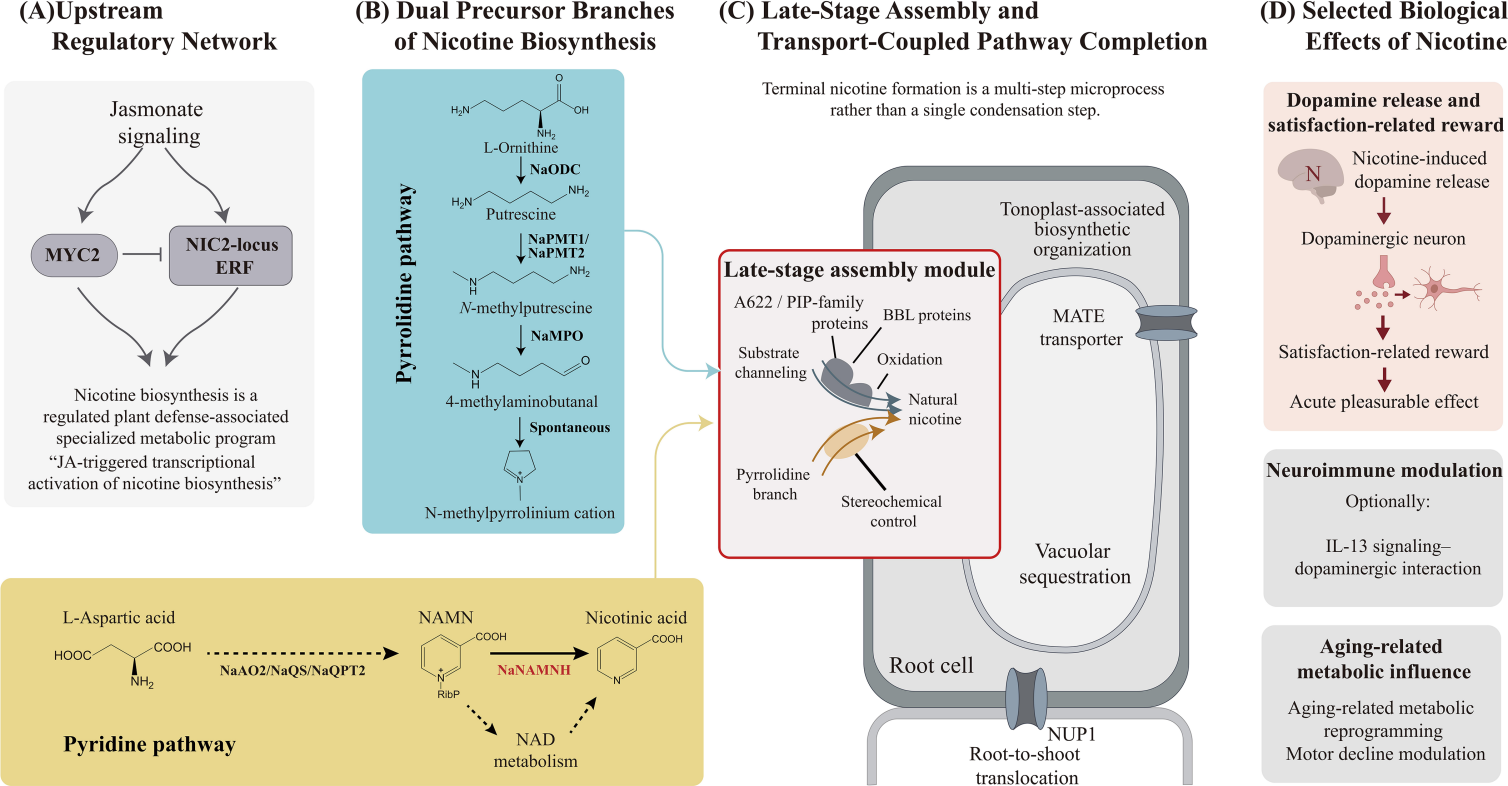
Conceptual framework of the complete nicotine biosynthesis and selected biological effects. **(A)** Jasmonate signaling activates the MYC2/NIC2-ERF regulatory module for nicotine biosynthesis; **(B)** Nicotine biosynthesis is supplied by pyridine and pyrrolidine precursor branches; **(C)** Late-stage nicotine formation involves multistep assembly, transport coupling, and compartmentation; **(D)** Selected downstream effects include dopaminergic reward, neuroimmune modulation, and aging-related metabolic influence.

Previous studies have elucidated much of the upstream logic of nicotine biosynthesis. The early steps of the pyrrolidine pathway, which starts from putrescine derived from either ornithine or arginine, are committed to alkaloid biosynthesis by putrescine N-methyltransferase and subsequent oxidation steps to form the N-methylpyrrolinium precursor (Dewey and Xie, 2013). In parallel, the pyridine branch was associated with the duplicated functions of NAD biosynthesis and the protein A622 was involved in the terminal steps of the pathway of some specialized alkaloid biosynthesis (Kajikawa et al., 2009, Kajikawa et al., 2017). Genetic studies have further demonstrated that phenotypes characterized by low nicotine levels are linked to clustered ERF regulators located at the NIC2 locus, while MYC2 serves as a connection between jasmonate signaling and pathway activation (**Figure 1A**). This suggests that nicotine biosynthesis is controlled by a coordinated defense regulon rather than by a collection of structural genes (Shoji et al., 2010; Shoji and Hashimoto, 2011). The pathway could explain precursor provisioning and late oxidation-related steps, but could not speak with testament to the decisive formation of natural nicotine. In particular, while BBL proteins were undoubtedly major determinants of the late pathway, their precise involvement in the scaffolding, oxidation, and stereochemical outcome was basically unresolved for many years (Kajikawa et al., 2011; Lewis et al., 2015; Vollheyde et al., 2023). This ambiguity meant that the pathway explained how plants prepare the building blocks for nicotine, but not fully how those building blocks are converted into the characteristic natural product.

The recent report on the complete biosynthesis of nicotine fundamentally alters the status of this field. This work is significant, not merely for adding another enzyme to an already crowded pathway map, but for completely changing the biosynthesis of nicotine from an extremely convincing, yet infrequently, partially biosynthesis model to a fully closed biosynthetic model system (Chang et al., 2026). The pathway that is ‘almost solved’ is essentially different from the pathway whose scaffold-forming logic has been completely and experimentally resolved and reconstructed. That distinction matters, because it allows nicotine to be reconsidered as a model for how plants organize complex nitrogen-containing specialized metabolites through coordinated chemistry, transport, and compartmentation, as summarized in **Figure 1** (Shoji et al., 2024; Chang et al., 2026).

## 3 The terminal step is not a single step

A useful implication of the new work is that the terminal stage of nicotine biosynthesis should no longer be considered as a single pathway (**Figure 1B**). Traditionally, the terminal reactions of nicotine synthesis have been described as condensation reaction and BBL model. However, the literature is already sparse but in empirical evidence-dense that the last terminal zone of the pathway as chemically and mechanistically less elaborate. The roles of A622/Phosphatidylinositolhosphate (PIP)-family proteins, BBL proteins, and late oxidation-associated reactions suggest that the terminal region of the pathway is chemically and mechanistically more complex than the traditional opinions (Kajikawa et al., 2009, Kajikawa et al., 2011). More recent chassis-engineering work in *Nicotiana benthamiana* also reinforced the importance of late-pathway control and stereochemical fidelity in nicotine-related alkaloid formation (Vollheyde et al., 2023).

While completing the pathway has been largely praised, there is also a more important lesson to be taken. What has been described in the literature as a missing step in specialized metabolism is not merely an undiscovered enzyme. Rather, the apparent absence in the step is more accurately described as a late stage microprocess involving some combination of substrate activation, selective channeling, a series of oxidative transformations, and stereochemical control. Nicotine biosynthesis now appears to align with this model. Although certain catalytic details may still require refinement, the understanding of the pathway has clearly progressed beyond a simplistic end-point condensation model. Moving forward, this should influence discussions and engineering of nicotine biosynthesis.

## 4 Transport and compartmentation are part of the biosynthetic solution

The latest report provides a new chance to assess the nicotine biosynthetic pathway (**Figure 1C**). Concerns about the ability of nicotine to synthesize and then move and be stored in certain areas of a plant have been largely dismissed. Previous studies showed that nicotine is synthesized predominantly in roots and then moved and sequestered through dedicated systems, including tonoplast-associated multidrug and toxic compound extrusion (MATE) transporters and nicotine uptake permease 1 (NUP1) (Morita et al., 2009; Kato et al., 2014). This also implies that the mobility of nicotine is tied to the metabolism of nicotine in different locations within the plant. Recent studies have shown a complete metabolism of nicotine and have associated the complete metabolism of the nicotine to the mobility and the presence of the biosynthetic systems within the tonoplast (Chang et al., 2026). In many biosynthetic pathways, transport is discussed as a downstream event that follows metabolite formation. However, the case of nicotine is different in that the storage and the transport of the metabolites are also considered to be a part of the formation of the metabolites. The pathway is best interpreted as a transport-coupled biosynthetic module rather than a simple series of soluble enzymatic reactions. This interpretation is also consistent with evolutionary work showing that nicotine biosynthesis emerged through duplication, recruitment, and regulatory integration of genes into a defense-associated network in tobacco (Kajikawa et al., 2017; Shoji et al., 2024). Once pathway closure is recognized, pathway architecture becomes at least as important as pathway inventory.

## 5 Multiple biological significance beyond the complete nicotine biosynthesis pathway

Because this article is centered on biosynthesis, the biological effects of nicotine are mentioned here to contextualize the importance of pathway completion. Nicotine crosses the blood-brain barrier and binds to nicotinic acetylcholine receptors, particularly within the nucleus accumbens, on dopaminergic neurons (**Figure 1D**). This firing of neurons facilitates the release of dopamine. This dopamine release and association of the nucleus accumbens with reinforcement and reward makes nicotine a powerful addictive chemical (De Biasi and Dani, 2011; McGranahan et al., 2011; Walker et al., 2023). Recent studies have shown that the nicotine reward and associated reinforcement can be significantly altered by inflammatory signaling. For example, in experimentally altered Interleukin-13 (IL-13) signaling, the reward associated with nicotine was markedly decreased as a result of reduced activity in the ventral tegmental area (Liu et al., 2026). In addition, long-term oral nicotine exposure has been reported to attenuate age-related motor decline in mice and to be associated with metabolic remodeling linked to sphingolipid homeostasis and NAD availability (Jia et al., 2025). Nevertheless, within the framework of plant specialized metabolism, these downstream effects remain secondary to the central mechanistic advance of resolving complete nicotine biosynthesis.

## 6 Conclusion

Most recent nicotine studies have little to no impact on the amount of annotation but rather on the understanding of the pathway mechanism. Defining the pathway has been a major accomplishment in solving late step chemistry, stereochemical control, transport, and subcellular organization. This has been a conceptual development in the field and suggests that nicotine biosynthesis should not be represented as an almost complete classical pathway with one elusive end step. Instead, it should be treated as a modern example of how plants build specialized alkaloids through integrated pathway architecture.

A completed pathway in one experimental framework does not automatically settle every species specific or ecological dimension of nicotine formation. It remains important to test how broadly the newly defined terminal logic is conserved across Nicotiana lineages, how flexible the transport-associated organization is under environmental perturbation, and which steps are genuinely flux limiting under native conditions. These are not weaknesses that diminish the recent advance, they are the next questions made possible by it. In conclusion, the complete biosynthesis of nicotine should be viewed as a reframing event for this field. It closes a longstanding mechanistic gap, elevates late-step organization and transport to central explanatory status, and turns nicotine into a high-value model for studying pathway completion in plant specialized metabolism.
